# Hallmarks of Cancer Expression in Oral Leukoplakia: A Scoping Review of Systematic Reviews and Meta-Analyses

**DOI:** 10.3390/cancers17152427

**Published:** 2025-07-22

**Authors:** Isabel González-Ruiz, Valerie Samayoa-Descamps, Karen Andrea Guagua-Cortez, Miguel Ángel González-Moles, Pablo Ramos-García

**Affiliations:** 1 Pius de Valls Hospital, 43800 Valls, Spain; 2 School of Dentistry, University of Granada, 18071 Granada, Spain; 3 Instituto de Investigación Biosanitaria Ibs.GRANADA, 18012 Granada, Spain

**Keywords:** oral leukoplakia, oral cancer, malignant transformation, cancer hallmarks, scoping review, systematic review, meta-analysis

## Abstract

Background/Objectives: Oral leukoplakia (OL) is a prevalent oral potentially malignant disorder. Despite its clinical relevance, the molecular basis of its progression to malignancy is not yet fully elucidated. This scoping review of systematic reviews and meta-analyses aimed to synthesize current knowledge and evidence gaps regarding the implications of hallmarks of cancer expression in OL malignant transformation. Methods: A systematic search was conducted in MEDLINE, Embase, DARE, and the Cochrane Library to identify systematic reviews (with or without meta-analysis) published up to April-2025. Results: Twenty-two systematic reviews were included. The most frequently explored hallmark was activation of invasion and metastasis (n = 12; 32.40%), followed by tumor-promoting inflammation (n = 10; 27.03%), evasion of growth suppressors (n = 8; 21.60%), sustained proliferative signaling (n = 3; 8.10%), energy metabolism reprogramming (n = 2; 5.40%), replicative immortality (n = 1; 2.70%), and resistance to cell death (n = 1; 2.70%). No evidence was found for angiogenesis or immune evasion in OL. Conclusions: Available evidence indicates that OL may develop oncogenic mechanisms in early stages of oral oncogenesis, especially those related to sustained proliferation, evasion of growth suppressor signals, and cellular migration and invasion. Chronic inflammation also may facilitate the acquisition of other hallmarks throughout the multistep process of oral carcinogenesis. These findings also reveal evidence gaps in underexplored hallmarks of cancer, which highlights the need to expand future primary- and secondary-level investigations to better define the molecular mechanisms underlying OL malignant transformation.

## Simple Summary

Oral leukoplakia (OL) is an oral potentially malignant disorder (OPMD) defined as a “predominantly white plaque of questionable risk having excluded (other) known diseases or disorders that carry no increased risk for cancer”, according to the latest consensus meeting of the WHO Collaborating Centre for Oral Cancer and Precancer. The molecular mechanisms involved in the malignant transformation of this relevant OPMD remain to be fully elucidated.

## 1. Introduction

In 2000, Hanahan and Weinberg [1] established the basis for a conceptual framework that changed our understanding of tumor biology by defining fundamental traits of neoplastic cells. According to their proposal, a cancer cell presents specific oncogenic features that are not acquired gradually but rather develop progressively through a multi-step process that begins in early, even premalignant, stages and culminates in the formation of a fully established tumor. It is now widely accepted that clonal expansion and selection of cell populations are central to this gradual acquisition of cancer hallmarks, a process largely driven by uncontrolled cell proliferation and genomic instability. The influence of Hanahan and Weinberg’s model has played a pivotal role in cancer research, inspiring over 130,000 studies that have applied this conceptual framework to investigate diverse dimensions of oncogenesis. This widespread impact led to an updated version published in 2011 [2], which incorporated new scientific insights from the subsequent decade. In this update, the authors consolidated six core hallmarks (sustained proliferative signaling, evasion of growth suppressors, resistance to cell death, replicative immortality, induction of angiogenesis, and activation of invasion and metastasis), alongside two enabling characteristics (genomic instability and tumor-promoting inflammation) and two emerging hallmarks (deregulation of cellular metabolism and evasion of immune destruction). This comprehensive model has significantly advanced our understanding of the molecular mechanisms underlying the initiation and progression of cancer and precancerous conditions, including those affecting the oral cavity.

In March 2020, an international group of experts in oral cancer were convened by the World Health Organization (WHO) Collaborating Centre for the Study of Oral Cancer in Glasgow, Scotland. The main objective of this international seminar was to update the nomenclature and classification of oral potentially malignant disorders (OPMDs), and to update rates of malignant transformation of OPMDs. As a result, a consensus document was released presenting the most robust and evidence-based findings to date on OPMD [3]. Oral leukoplakia (OL) is considered the most relevant OPMD affecting the oral mucosa. It is defined as a “predominantly white plaque of questionable risk having excluded (other) known diseases or disorders that carry no increased risk for cancer” [3]. Today it is estimated that 1.36% to 2.60% of the global population is affected by OL [4,5,6]. The OL malignant transformation rate is 6.64%, derived from the results of the most recent meta-analysis that has been published on the subject [7]. The risk factors that most influence the malignancy rate are the non-homogeneous clinical types, larger size lesions, located on the lateral margin of the tongue and tobacco use, as well as histopathological parameters (i.e., presence and degree of epithelial dysplasia) [7]. Regarding the molecular landscape underlying the malignant transformation of OL, it has been proposed that this OPMD reflects an epithelial condition mainly characterized by cell proliferation, the disruption of tumor suppressor pathways, failure of apoptotic control, and the progressive accumulation of genomic instability [8]. However, the precise oncogenic drivers and molecular biomarkers implicated in this transformation remain largely elusive. Moreover, the potential involvement of emerging hallmarks of cancer—such as chronic inflammation, replicative immortality, or immune evasion—is not yet fully understood in this OPMD. We hypothesize that the malignant progression of OL does not result from the isolated activation of a single molecular mechanism, but rather from the synergistic interplay among multiple pathways, encompassing canonical and emerging hallmarks of cancer. A better understanding of these mechanisms may offer novel translational insights into the risk stratification, monitoring and creating new opportunities for targeted therapeutic interventions in patients with OL. Given this contextual framework, the present scoping review of systematic reviews and meta-analyses was designed to explore and synthesize the existing evidence on the expression of cancer hallmarks in the malignant transformation of oral leukoplakia. This study also aims to inform future evidence-based research directions by identifying current knowledge gaps in this topic.

## 2. Materials and Methods

This scoping review was conducted following the reporting guidelines of the Preferred Reporting Items for Systematic Reviews and Meta-Analyses extension for Scoping Reviews (PRISMA-ScR) [9]. A study protocol was registered on Open Science Framework (OSF) and is publicly accessible (registration DOI: 10.17605/OSF.IO/FQH86; link: https://osf.io/utghf/). OSF is an open source platform where researchers can share protocols, data, and contribute to transparency of research.

### 2.1. Search Strategy

A thorough systematic search was performed across several databases, including the Cochrane Database of Systematic Reviews (Cochrane Library), the Database of Abstracts of Reviews of Effects (DARE), MEDLINE (via PubMed), and Embase, covering publications of secondary-level studies up to April-2025. No restrictions were set for the earliest date of inclusion. The full search strategy, designed in line with the PRESS recommendations [10], is available in Supplementary Materials (Table S1). It was designed combining controlled synthax (e.g., MeSH and EMTREE terms) with free-text terms to maximize sensitivity (Table S2, in Supplementary Materials). The primary keywords included “oral leukoplakia,” combined with terms related to cancer hallmarks, biomarkers, and oncogenic processes. Gene and protein terms were classified according to their functional roles within the hallmarks of cancer frameworks. Synonyms and functionally equivalent genes/proteins, including those referenced in the original Hanahan and Weinberg publication [1,2], were verified using the HUGO Gene Nomenclature Committee database (https://www.genenames.org). The search syntax was further optimized using a validated filter from the Centre for Reviews and Dissemination (CRD), specifically designed to retrieve systematic reviews and meta-analyses [11,12]. To enhance completeness, additional studies were located by manually screening the reference lists of the included reviews and by conducting additional searches in Google Scholar. Reference management was carried out using Mendeley v.1.19.8 (Elsevier, Amsterdam, The Netherlands), and duplicate records were eliminated prior to the screening process.

### 2.2. Eligibility Criteria

Systematic reviews—regardless of whether meta-analysis was performed—were considered eligible if they examined cancer hallmarks, biomarkers, or oncogenic pathways or mechanisms associated with the malignant transformation of OL. For this study, a “systematic review” was defined as one that explicitly stated a research question and employed systematic, transparent methods, at a minimum including a search strategy and predefined eligibility criteria, to identify, select, critically assess, and synthesize data from primary level studies. No limitations were imposed based on language, publication date, or other factors such as geographical region, sex, or age of patients.

### 2.3. Study Selection Process

Reviewers independently applied the eligibility criteria. Disagreements were discussed with a supervising author and resolved by consensus. Articles were selected in two phases. First, titles and abstracts were screened to identify articles that met the inclusion criteria. Then, the full texts of the selected articles were reviewed, and those that did not meet the eligibility criteria were excluded. Any discrepancies were resolved through consensus.

### 2.4. Data Extraction

Coauthors independently extracted data from the included studies using a standardized collection template. The process was conducted with Microsoft Excel and Word (v.16/2018, Microsoft, Redmond, WA, USA). Information retrieved included first author, year of publication, target population (i.e., oral leukoplakia), sample size (i.e., number of studies reviewed), type of study (systematic reviews with/without meta-analyses), biomarkers, oncogenic mechanisms and/or hallmarks of cancer assessed, and main analysis results. All data entries were verified through multiple cross-checking rounds, and any inconsistencies were resolved by consensus.

### 2.5. Evaluation of Risk of Bias and Quality of Evidence

After the data extraction process, we collected and documented the tools and methods employed in each included systematic review to critically assess the risk of bias and quality of evidence. This included the identification of standardized instruments to critically judge observational primary-level studies or specific investigations about tumor biomarkers, such as the Newcastle–Ottawa Scale (NOS) tool [13], Quality in Prognosis Studies (QUIPS) tool [14], or REporting recommendations for tumor MARKer prognostic studies (REMARK) statement [15]. In addition, we recorded the methodological and reporting guidelines followed by systematic reviews, e.g., Preferred Reporting Items for Systematic reviews and Meta-Analyses (PRISMA) reporting guidelines [16], Joanna Briggs Institute (JBI) resources [17], or Cochrane guidance directions [18]. Finally, we identified whether the systematic reviews applied any formal approach to rate the overall quality or certainty of evidence, such as the Grading of Recommendations Assessment, Development and Evaluation (GRADE) system [19]. The presence, type, and findings of these assessments were extracted and analyzed descriptively to explore how risk of bias and quality of evidence were evaluated and reported within the included secondary-level studies.

### 2.6. Rationale, Critical Analysis, and Evidence Synthesis

The rationale of a scoping review is to explore the existing evidence on a topic, identify gaps in knowledge, and guide future research directions, following internationally accepted methodological frameworks and consensus guidelines that endorse its use when the objective is to provide a comprehensive synthesis of a heterogeneous and complex evidence base [9,20,21]. The purpose of this scoping review was to explore whether systematic reviews—with or without meta-analysis—focusing on patients with oral leukoplakia have examined the hallmarks of cancer, as well as related biomarkers and oncogenic mechanisms, in the context of their potential role in malignant transformation. The objective was to synthesize the available evidence and identify gaps in the current literature. Results were presented using descriptive tables and figures. The findings underwent thorough and critical evaluation within a structured methodological approach, and finally were discussed in depth.

## 3. Results

### 3.1. Results of the Literature Search

**Figure 1:**
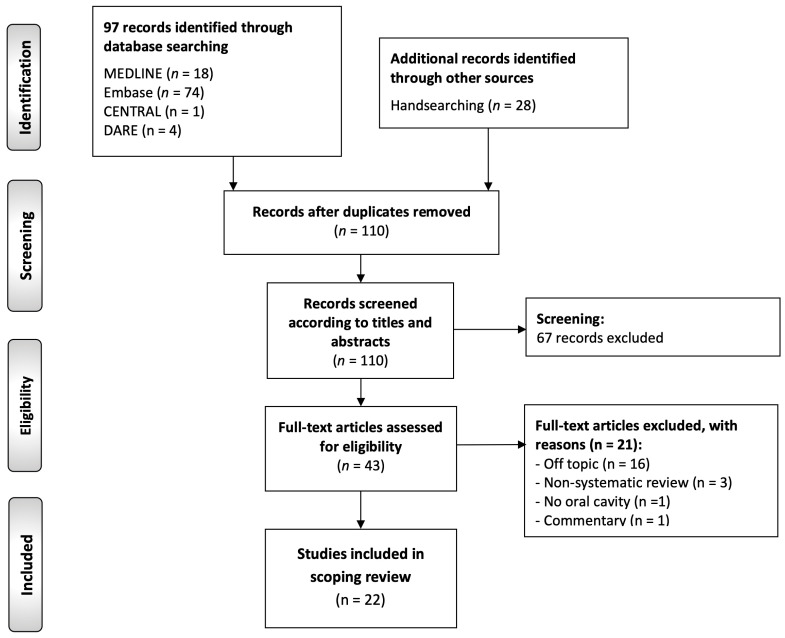
Flow diagram of the identification and selection process of the studies included in this scoping review of systematic reviews and meta-analyses.

The main results of the search strategy are illustrated in Figure 1, which includes the identification and selection of studies incorporated in this scoping review. A total of 125 records were retrieved, 74 from Embase, 18 from MEDLINE (via PubMed), 4 from DARE, 1 from CENTRAL, and 28 from manual searches, primarily through Google Scholar and reference list screening. After removing duplicates, 110 records were eligible for preliminary screening based on titles and abstracts. This process yielded a subset of 43 records that were provisionally included and underwent full-text review. Following a detailed evaluation, 21 records were excluded (the full-text excluded studies and their reasons for exclusion can be found in List S2), resulting in a final sample of 22 secondary-level studies [22,23,24,25,26,27,28,29,30,31,32,33,34,35,36,37,38,39,40,41,42,43]. All included studies were systematic reviews, with or without meta-analyses, fulfilling all predefined inclusion criteria for critical appraisal and evidence synthesis in this scoping review.

### 3.2. Study Characteristics

Table 1 summarizes the characteristics of the study sample (n = 22), of which the majority included meta-analyses (n = 14; 63.63%), while the remainder did not (n = 8; 36.36%). The earliest study was published in 2019, and the most recent in 2025. All studies investigated oral leukoplakias, either alongside other potentially malignant oral disorders (n = 14; 63.63%) or as a distinct clinical entity (n = 8; 36.36%).

### 3.3. Risk of Bias and Quality of Evidence

We recorded the critical appraisal tools used by each included systematic review to evaluate risk of bias (Table 2). All included reviews applied standardized risk of bias assessment tools (n = 22, 100%), as this was one of the predefined eligibility criteria of the present scoping review. The most frequently used tool was the Quality in Prognosis Studies (QUIPS) tool, developed by Cochrane Prognosis Methods Group; this tool is universally used in systematic reviews investigating prognostic factors (n = 9, 40.9%); followed by the Newcastle–Ottawa Scale (NOS) tool (n = 6, 27.3%), originally developed by the University of Newcastle and the University of Ottawa, this tool is widely used in systematic reviews for assessing the quality of non-randomized studies, particularly observational cohort designs. In contrast, only one study assessed the quality or certainty of the evidence using standardized approaches (i.e., GRADE system) (Table 2). Overall, a wide range of instruments were employed, reflecting a source of methodological heterogeneity, which in turn is logical, given the lack of international consensus on the choice of these tools and the large arsenal of excellent methods for assessing the risk of bias at our disposal, as well as the absence of universal consensus regarding the most appropriate quality of evidence tool for prognosis-focused systematic reviews. Nevertheless, all tools identified in this review are widely accepted and considered robust frameworks for critical appraisal.

### 3.4. Critical Analysis and Evidence Synthesis

The biomarkers explored in the included systematic reviews are summarized in Table 3, organized according to the original cancer hallmark framework proposed by Hanahan and Weinberg. The most frequently studied proteins were p53 and podoplanin, each analyzed in five systematic reviews. Interleukin-6 and TNF-α followed, each examined in three studies. ALDH1, LDH, interleukin-1β, and CD133 were each investigated in two studies, respectively. Thirteen additional proteins were each evaluated by a single study, including Cyclin D1, EGFR, Ki-67, pRb, p27, p16, CYFRA21, BMI1, CEA, β-catenin, E-cadherin, Twist, and MMP9.

**Figure 2:**
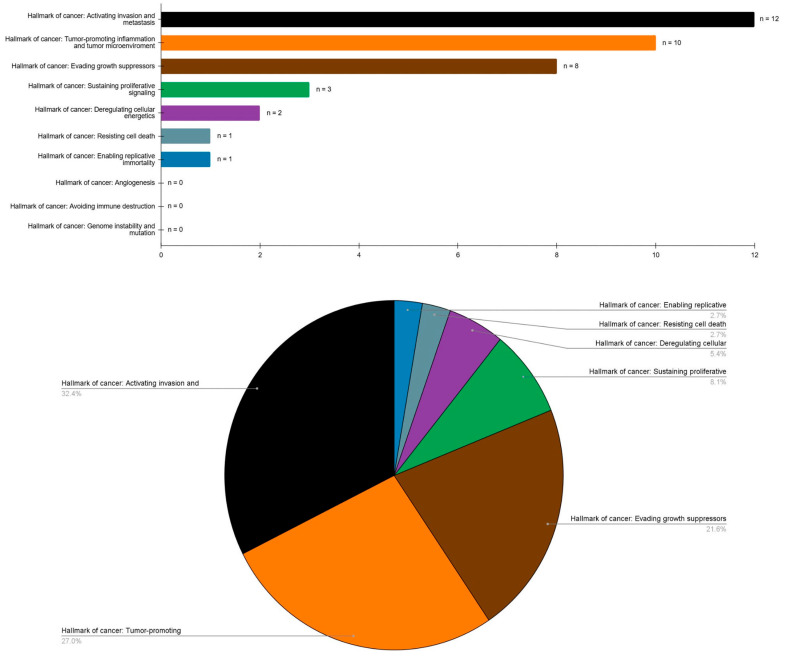
Bar and pie charts graphically summarizing the evidence derived from secondary-level systematic reviews and meta-analyses on the hallmarks of oral cancer in oral leukoplakia malignant transformation. Hallmarks of cancer are shown ordered by absolute counts (top, bar chart) and by relative frequencies as raw proportions expressed as percentages (bottom, pie chart).

Key study characteristics and findings, categorized by biomarkers and their corresponding cancer hallmarks, are synthesized in Table 4. Furthermore, counts and relative frequencies are graphically depicted in Figure 2. The most frequently analyzed hallmark was activation of invasion and metastasis, addressed in 12 studies (n = 12; 32.40%), followed by tumor-promoting inflammation (n = 10; 27.03%), evasion of growth suppressors (n = 8; 21.62%), and sustained proliferative signaling or uncontrolled proliferation (n = 3; 8.10%). Less frequently investigated hallmarks included deregulation of cellular energy metabolism (n = 2; 5.40%), resistance to cell death (n = 1; 2.70%), and replicative immortality (n = 1; 2.70%). In contrast, clear evidence gaps were identified for angiogenesis, evasion of immune destruction, and genomic instability and mutation, none of which were addressed by the included secondary-level studies (n = 0; 0%).

The results of this scoping review revealed that a limited but consistent group of molecular mechanisms has been preferentially investigated in relation to OL malignant transformation (the main molecular mechanisms are listed in Table 5). Sustained proliferative signaling biomarkers showed promising results, particularly through the evaluation of EGFR overexpression (RR  =  1.85, 95% CI = 1.31–2.59; *p* < 0.001) and *CCND1*/cyclin D1 upregulation (RR = 1.86, 95% CI = 1.13–3.06; *p* = 0.01), both showing a large effect size in meta-analyses. Tumor suppressors also showed interesting results, specifically the proteins p53 (RR = 2.22, 95% CI = 1.35–3.64, *p* = 0.002), pRb (RR = 2.00, 95% CI = 1.22–3.29, *p* = 0.006), and p16 (RR = 2.01, 95% CI = 1.3–2.96; *p* < 0.001), all showing a quite similar magnitude of association. Another mechanism with high predictive value was the activation of cellular migration and invasion, particularly mediated by podoplanin overexpression (HR = 3.72, 95% CI = 2.40 to 5.76; *p* < 0.001), showing the highest effect size in quantitative meta-analytical terms. Finally, tumor-promoting inflammation was also identified as a relevant enabling mechanism, specifically, the presence of proinflammatory cytokines such as IL-6 (SMD = −1.07, 95% CI = −1.86 to −0.28, *p* = 0.008) or TNF-α. (SMD = −0.83, 95% CI = −1.61 to −0.05, *p* = 0.04), which demonstrated progressively increasing expression profiles in leukoplakia compared to normal oral mucosa. Overall, these mechanisms and biomarkers represent the most robust and promising molecular alterations currently described in the secondary-level literature regarding OL malignant transformation (Table 4 and Table 5).

## 4. Discussion

### 4.1. Maintenance of Proliferative Signaling

Under physiological conditions, oral epithelial proliferation is tightly regulated to maintain tissue structure and function. In contrast, sustained and uncontrolled hyperproliferation is a hallmark of cancer, closely linked to the acquisition of genomic instability [1,2,44]. The hyperproliferative state facilitates the accumulation of mutations in oncogenes and tumor suppressor genes, and promotes clonal expansion of malignant cells, ultimately driving the progression toward oral cancer [1,2,44]. The involvement of sustaining proliferative signaling pathways in OL has been explored in three systematic reviews with meta-analyses, all of which assessed key proliferation-related biomarkers, including cyclin D1, EGFR, and Ki-67 [27,30,37]. EGFR, frequently overexpressed in human tumors [45,46,47,48], triggers MAPK and PI3K/Akt/mTOR pathways that drive proliferation via ligand-dependent or constitutive activation [15,49,50,51,52,53,54,55,56]. EGFR upregulation was significantly associated with an elevated OL malignant transformation rate (RR = 1.85, 95% CI = 1.31–2.59, *p* < 0.001) [37]. On the other hand, cyclin D1 (encoded by *CCND1*, which frequently shows gene amplification in head and neck cancers) is a key downstream effector of EGFR signaling and associated pathways, promoting cell cycle progression and contributing to the hyperproliferative phenotype of OL [15,55,56]. *CCND1*/cyclin D1 upregulation was found to be significantly associated with an increased risk of malignant transformation in OL (RR = 1.86, 95% CI = 1.13–3.06, *p* = 0.01) [30]. Finally, the expression of Ki-67—a proliferation marker commonly used in pathology laboratories to indicate active cell proliferation—progressively increased from normal mucosa to OL and oral cancer (*p* < 0.001) [27]. Some authors have suggested that OL patients overexpressing Ki-67 may have a higher risk of OSCC development. Although proliferative mechanisms have traditionally been regarded as the most critical drivers of malignant transformation in OL, it is increasingly evident that they do not act in isolation. Instead, their oncogenic potential is likely amplified through interplay with other cancer hallmarks, such as evasion of growth suppressors, inflammation, and tissue invasion. Future studies should aim to assess the predictive value of biomarker panels that reflect this synergistic behavior, rather than evaluating isolated markers in a fragmented manner.

### 4.2. Evasion of Growth-Suppressive Signals and Development of Resistance to Cell Death

The tumor suppressor protein p53 (also known as the guardian of the genome), encoded by the *TP53* gene, plays a central role in maintaining genomic integrity through its involvement in cell cycle arrest, apoptosis, and DNA repair [57,58,59]. Its relevance in the context of OL has been supported by two systematic reviews with meta-analytical approaches [27,29], which have consistently highlighted its association with malignant progression in this OPMD. Specifically, one of these studies demonstrated that p53 overexpression significantly associates with an increased risk of malignant transformation in OL (RR = 2.22; 95% CI: 1.35–3.64; *p* = 0.002) [29]. Additionally, another meta-analysis reported a progressive elevation in p53 expression levels along the progression from normal oral mucosa to leukoplakia and ultimately to OSCC, emphasizing its potential role as an early biomarker of neoplastic evolution in OLs [27]. The loss of key tumor suppressor proteins involved in cell cycle regulation has been increasingly recognized as a critical event in the progression of OL toward malignancy. A systematic review and meta-analysis has provided secondary-level evidence supporting the association between the loss of expression of the retinoblastoma protein (pRb)—which exerts its tumor suppressor function by binding and inactivating E2F transcription factors, thereby blocking entry into the S phase and preventing uncontrolled cell cycle progression [60,61,62,63,64,65,66]—and a significantly elevated risk of malignant transformation in OL (RR = 2.00; 95% CI: 1.22–3.29) [42]. This finding points out the pivotal role of the RB pathway in early tumor suppression in patients affected by OL on the road to malignancy. Similarly, another meta-analytic study focusing on *CDKN2A*, which encodes the p16 protein [58] and which acts as a key tumor suppressor by inhibiting cyclin-dependent kinases, thereby enforcing cell cycle arrest at the G1 phase, revealed that increased expression of p16 is also significantly linked to a higher risk of malignant transformation (RR = 2.01, 95% CI = 1.36 to 2.96; *p* < 0.001) [43], suggesting a possible compensatory upregulation in response to oncogenic stress or disrupted regulatory circuits. In addition, a systematic review highlighted p27, another cyclin-dependent kinase inhibitor, as a promising prognostic biomarker for malignant progression in OL, although this evidence has not yet been supported by quantitative meta-analysis [36], which reinforce the need to further investigate the involvement of this biomarker in OL malignant transformation. In summary, the findings of this scoping review suggest the loss of tumor suppressor function and resistance to apoptosis—mainly via p53, pRb, and p16 oncogenic mechanisms—favors a sustained hyperproliferative state, facilitating the acquisition of genomic instability, with persistence and expansion of malignant clones within the OL oral epithelium. Future research should aim to validate the predictive role of tumor suppressor proteins such as p53, pRb, p16, and p27 through well-designed prospective studies, ideally integrating them into multimarker panels. On the other hand, to date no systematic reviews or meta-analyses have addressed other relevant pathophysiological processes, such as autophagy [67,68,69], necrosis [70,71,72,73], other apoptosis-related mechanisms (linked to proapoptotic-Bax, Bak, TNF, FAS-L, TRAIL [67,74,75,76,77,78], or anti-apoptotic-Bcl-2 or Bcl-xL [76,77] regulators), other relevant tumor suppressor signaling pathways (e.g., TGF-β/Smad-dependent signaling pathway [79,80,81,82,83,84,85]), the loss of contact inhibition phenomenon [86,87,88,89,90,91,92], or the consequences of the dysregulation of these alterations in the malignant transformation of OL. These oncogenic mechanisms, despite being well-established in the biology of cancer, remain entirely unexamined within the context of OL. There is a critical need to explore the implications of these uninvestigated oncogenic processes in OL malignant transformation. Addressing these evidence gaps could uncover novel biomarkers and therapeutic targets for early intervention in patients with this OPMD.

### 4.3. Enabling Replicative Immortality

Telomere shortening limits cell divisions to prevent aging and maintain genomic stability under physiological conditions [93,94]. Critically short telomeres trigger cell death, often through TP53-dependent mechanisms [95,96]. However, if tumor suppressors fail, early neoplastic clones may evade this barrier. Telomerase reactivation allows these clones to maintain telomere length, promoting survival and proliferation. Its activity is commonly found in OSCC and linked to resistance to apoptosis and genomic stabilization in advanced stages; nevertheless, most of the available evidence on this hallmark originates from studies conducted in oral cancer and other malignant neoplasms [15,97,98], which are beyond the scope of the present scoping review. Only one systematic review and meta-analysis [32] has been published on this hallmark, specifically focused on the biomarker Bmi1, which was analyzed together with ALDH1 and CD133. The subgroup meta-analysis for OL showed that this combination of biomarkers was significantly associated with higher risk of malignant transformation (RR = 3.19, 95% CI = 2.55–3.98). BMI is a member of the complex PCR1 (Polycomb repressive complex 1), with potential implications in gene silencing by regulating chromatin structure. It can suppress tumor suppressor proteins and pathways (e.g., p16, pRb, p53, MDM2), thereby allowing progression through the cell cycle. It is essential for the maintenance and self-renewal implicated in mediating cellular senescence. Although some of the functions discussed relate to replicative immortality, given the pleiotropic nature of BMI, future studies are needed to provide more evidence on this hallmark of cancer in OLs. We should note that significant evidence gaps remain regarding this cancer hallmark and its relationship with the malignant transformation of OL. Notably, telomerase activity, the key enzyme involved, has not yet been investigated in this context. Similarly, its catalytic subunit, hTERT, which is commonly assessed through immunohistochemical technique, has not been studied in relation to OL progression. Therefore, it is imperative that future research explores these biomarkers, given their potential role in preserving telomere length, preventing telomere attrition, and enabling epithelial cells within OLs to acquire replicative immortality.

### 4.4. Induction of Angiogenesis

Angiogenesis, the formation of new blood vessels from pre-existing vasculature, plays a crucial role in tumor progression by providing nutrients, oxygen, and a pathway for metastasis [2]. In the context of oral leukoplakia (OL), this hallmark of cancer may represent a key event in the transition from a premalignant lesion to invasive carcinoma [99]. However, to date, there is a remarkable absence of systematic reviews or meta-analyses specifically addressing the role of angiogenesis in OL malignant transformation. This gap highlights the current limitations in the high-level evidence available for this mechanism within the scope of oral potentially malignant disorders. It is important to emphasize that the objective of the present scoping review is not to analyze individual primary studies on angiogenesis markers. Including such data would conflict with the defined methodology and could compromise the objectivity and balance of this study. Given this evident shortfall, future investigations should prioritize the evaluation of angiogenesis-related biomarkers in OL, particularly vascular endothelial growth factor (VEGF) and its receptor (VEGFR) [100], the most investigated proangiogenic factors in human carcinogenesis. Well-designed primary studies and subsequent meta-analyses are essential to clarify their predictive value in OL malignant transformation and their potential use in early risk stratification.

### 4.5. Activation of Invasion and Metastasis

Metastasis has traditionally been considered a late event in advanced cancer disease [2,101], although current evidence shows that its molecular regulators are active even in early stages of human carcinogenesis [102]. This shift highlights the relevance of studying migration and invasion pathways from the onset of tumor development in OPMDs. In this sense, several systematic reviews with meta-analysis have been published on the expression of proinvasive markers in OL and their implications in malignant transformation, and a wide spectrum of molecules were explored (podoplanin, β-catenin, E-cadherin, Twist, CEA, ALDH, and MMP-1) [22,24,25,26,31,32,34,35,36]. Most of them are regulators of epithelial–mesenchymal transition (EMT), a phenomenon which enables epithelial cells to acquire migratory and invasive capabilities by remodeling adhesion and cytoskeletal structures [103,104,105,106,107]. In this context, podoplanin—a regulator of actin cytoskeleton dynamics, promoting cell motility and invasion during EMT—has received the greatest attention in recent published systematic reviews, and is currently considered the most promising marker of this hallmark in the prediction of malignant transformation of OLs [24,25,31,34,36]. Only a single systematic review has confirmed with meta-analytical techniques its predictive value, demonstrating a high predictive capacity for malignant transformation of OLs (HR = 3.72, 95% CI = 2.40–5.76, *p* < 0.001) [25]. Interesting promising results were also reported for E-cadherin, β-catenin, Twist, and MMP-9; however, these secondary-level studies did not apply meta-analytical techniques, so future research is needed to demonstrate their implications in malignant transformation of OLs on the basis of higher evidence. Future research should aim to fill the substantial evidence gaps regarding the role of key transcription factors involved in EMT phenomenon, such as Snail, Slug, and ZEB1/2. Although these molecules are well-established regulators of cell motility and invasiveness, their specific implications in the early stages of OL progression remain poorly understood. Moreover, given the consistent association of podoplanin with increased migratory potential and malignant transformation in OL, it would be highly valuable to explore its combined predictive performance with other molecular biomarkers. This integrative approach could help refine risk stratification, identify high-risk lesions more accurately, and, perhaps, guide the development of personalized therapeutic strategies.

### 4.6. Enabling Characteristics

Hanahan and Weinberg also emphasized that the defining hallmarks of cancer are progressively acquired by neoplastic cells, enabled by certain underlying conditions [2]. Among the most significant of these enabling factors are genomic instability—characteristic of both premalignant and malignant cells—and a tumor-promoting inflammation, commonly present in precancerous lesions or surrounding tumors, which, as we will explore, plays an active role in promoting oncogenesis [2].

Current knowledge regarding genomic instability in leukoplakias is largely based on indirect evidence derived from the study of other interconnected cancer hallmarks, such as sustained proliferative signaling, evasion of growth suppressors, and tumor-promoting inflammation, whose secondary-level evidence from systematic reviews and meta-analyses has already been discussed [27,29,30,37,42,58]. These processes are intimately associated with the accumulation of genetic alterations and chromosomal abnormalities that drive malignant transformation [15]. Genomic instability is often activated by hyperproliferation, allowing for the clonal expansion of cells with oncogenic mutations in tumor suppressor genes [108], or dysfunction in caretaker genes [109]. In oral cancer and OPMD specifically, recurrent amplifications of chromosomal regions like 3p26 and 11q13 lead to overexpression of oncogenes such as *CCND1*, reinforcing proliferative signaling and further promoting genomic instability [15]. Future research should directly investigate this hallmark of cancer in oral leukoplakia. High-priority areas include the identification of specific chromosomal alterations and mutations in caretaker genes such as BRCA1/2 [110]. Additionally, the frequency and impact of genomic amplifications (e.g., 3p26 and 11q13) should be explored in premalignant lesions. Studies combining genomic profiling with clinical outcomes could help validate biomarkers of early malignant potential. These efforts are essential to clarify the role of genomic instability in the earliest stages of oral carcinogenesis.

Chronic inflammation is now recognized as a key cancer enabler, present in tumors and premalignant lesions. Rather than just an immune response, inflammatory infiltrates promote oncogenesis via chemokines and growth factors [72,111,112,113]. Regarding our current understanding of the role that chronic inflammation plays in the malignant transformation of OL, several secondary-level studies have been published [23,28,31,32,38,39], evaluating a set of inflammatory markers such as IL-6, IL-1β, TNF-α, and CD133. Meta-analytical studies point out that all cytokines studied showed significantly higher expression levels in OL than in healthy controls, and their expression was also significantly increased in cases of oral cancer [23,28,38,39]. Despite the recognized relevance of tumor-promoting inflammation in oral carcinogenesis, significant knowledge gaps remain unexplored in leukoplakias. Although it has demonstrated relevance in cancer-related inflammation, the NF-κB pathway remains virtually unknown. Considering that NF-κB controls the transcription of key inflammatory mediators, survival factors, and oncogenes, its dysregulation could have a significant impact on OL progression; therefore, more research is needed on this topic. Another essential research direction is to determine whether tumor-promoting inflammation is common to all OLs or varies between clinical subtypes. It is plausible that erythroleukoplakias, known for their higher dysplasia rates and malignant potential, present stronger activation of pro-inflammatory and pro-survival signaling compared, for example, to homogeneous leukoplakias. Such subtype-specific differences could have both diagnostic and prognostic implications. Future studies should systematically stratify OLs based on their clinical presentation when assessing inflammatory signaling. This approach would help refine risk stratification and personalize follow-up strategies.

### 4.7. Reprogramming of Energy Metabolism

The malignant transformation of leukoplakias involves not only genetic and molecular alterations but also relevant metabolic reprogramming that enables abnormal cell growth and survival. One of the most relevant shifts observed in cancer biology is the adaptation of energy metabolism to support the increased biosynthetic demands of proliferating cells [2]. Recent systematic reviews with meta-analyses have provided secondary-level evidence on the potential role of lactate dehydrogenase (LDH) as a metabolic biomarker in OL. LDH, a key enzyme in the glycolytic pathway, catalyzes the conversion of pyruvate to lactate under anaerobic conditions, and its overexpression has been linked to altered metabolic states in neoplastic cells. Quantitative analysis has demonstrated that LDH expression is significantly higher in OL tissues compared to normal oral mucosa (SMD = 11.67, 95% CI = 1.01 to 22.33; *p* = 0.03), while being lower than in oral squamous cell carcinomas (SMD = 5.62, 95% CI = 2.14 to 9.11; *p* = 0.002) [40,41]. These findings suggest that LDH may serve as an intermediary metabolic marker in the stepwise progression from normal epithelium to cancer, offering possible value in early detection strategies. These observations align with the well-established Warburg effect, a metabolic hallmark of cancer described nearly a century ago, in which cells preferentially engage in aerobic glycolysis even in the presence of oxygen [114,115,116,117]. While less efficient in ATP production compared to oxidative phosphorylation, this pathway supports the anabolic requirements of rapidly dividing cells by supplying intermediates for nucleotide, lipid, and amino acid synthesis [114,115,116,117]. The upregulation of LDH in OL may reflect this early metabolic reprogramming, supporting the survival and expansion of dysplastic epithelial clones. Despite these promising findings, some knowledge gaps remain. It is still unclear whether LDH upregulation is a driver or merely a byproduct of transformation in OL. Future studies should also explore additional metabolic enzymes involved in glycolysis, investigate their expression in different OL subtypes, and assess their combined predictive value for malignant progression.

### 4.8. Evading the Antitumor Immune Response

Immune evasion, the ability of tumor and premalignant cells to escape immune surveillance, has emerged as a critical hallmark of cancer. In leukoplakias, this mechanism may enable epithelial cells to resist immune-mediated elimination, thereby contributing to malignant progression [118,119,120]. Nevertheless, in a similar way to what happened in the hallmark angiogenesis and despite its biological relevance, no systematic reviews or meta-analyses have yet assessed the role of immune evasion—particularly through the PD-L1/PD-1 pathway—in the malignant transformation of this relevant OPMD. This highlights a notable gap in secondary-level evidence on this emerging oncogenic mechanism. It is important to clarify again that the present scoping review does not aim to provide or analyze primary-level findings on immune evasion biomarkers. Including such results would exceed the methodological scope of this study and potentially compromise the scientific neutrality and integrity of the present study. Given the limited high-level evidence, future research should explore the prognostic value of immune checkpoints such as PD-L1 and PD-1 in OL. New studies should aim to clarify whether their overexpression marks a true immunosuppressive mechanism facilitating transformation, and whether these pathways could serve as predictive biomarkers in patients with high-risk OLs or as future potential therapeutic targets.

### 4.9. Study Limitations and Potential Strengths

Some limitations should be discussed. First, methodological heterogeneity was identified across systematic reviews in the choice of specific risk of bias tools (QUIPS tool, NOS tool, etc.), reflecting the absence of an international consensus on a single standard and the availability of multiple robust frameworks widely accepted and considered reliable for critical appraisal. Additionally, It should be noted that the majority of systematic reviews rigorously controlled for selection bias (i.e., pertinent diagnostic criteria were applied in the primary-level studies to confirm the diagnosis of OL, ensuring that study samples truly represent the target populations). In contrast, a potential risk of bias related to the control of confounding factors was identified across these systematic reviews. This limitation is typically the Achilles’ heel of observational studies, which often fail to collect all relevant clinicodemographic variables (e.g., sex, age, tobacco and alcohol use, educational and socioeconomic status, among others) and do not perform appropriate multivariable statistical adjustments to evaluate the impact of these factors on the prognostic outcomes investigated. Second, relevant evidence gaps were found for key hallmarks of cancer, such as the hallmark induction of angiogenesis or the hallmark evading the antitumor immune response, which still require further primary-level and secondary-level studies on their oncogenic implications in the malignant transformation of leukoplakias. This disparity in the distribution of evidence highlights the need to focus future research efforts on less explored areas.

As potential strengths, current knowledge as well as evidence gaps on the hallmarks of cancer in OL have been comprehensively explored, and a broad spectrum of carcinogenesis mechanisms has been discussed. In light of the results obtained in this scoping review and the available secondary-level evidence, we would like to propose a molecular hypothesis regarding the main oncogenic mechanisms involved in the malignant transformation of oral leukoplakia (listed alongside their oncogenic roles in Table 5), in order to provide greater originality and added scientific value. Among these, the overexpression of EGFR stands out; together with the activation of its associated intracellular signaling pathways (MAPK and PI3K/Akt/mTOR) and the overexpression of cyclin D1, it is promoted as a hyperproliferative oncogenic signaling significantly associated with increased risk of OL malignant transformation. Another molecular mechanism that appears to be critical in determining the poor prognosis of OL is the loss of tumor suppressor gene function, particularly TP53, RB, and CDKN2A. Specifically, TP53 gene mutation and/or overexpression of its protein product p53 are alterations primarily linked to a reduction in the ability to repair damaged DNA and to increased resistance to apoptosis. In parallel, functional alterations of pRb and p16 contribute to the inhibition of tumor growth suppression. The loss of these key tumor suppressors favors the persistence of potentially malignant clones within the oral epithelium of OLs. Another relevant mechanism is the activation of tissue migration and invasion, traditionally associated with advanced stages of carcinogenesis, but now increasingly recognized as an early event in OL. In this regard, podoplanin overexpression has shown a strong predictive capacity for malignant transformation in OL and currently represents one of the most promising markers according to the available secondary-level evidence. In addition to these mechanisms, the role of chronic inflammation must also be highlighted, particularly cytokines such as IL-6, IL-1β, and TNF-α, which generate a pro-oncogenic microenvironment that favors uncontrolled proliferation and the acquisition of genomic instability, an oncogenic state that may further drive new molecular alterations in OL. We believe that the interaction among these mechanisms, as included in this molecular hypothesis of OL malignant transformation, creates a fertile ground for the development of oral cancer in the affected epithelium of patients with this OPMD. Malignant transformation in OL likely does not occur through the isolated activation of any single mechanism, but rather through the synergy among several of them, which could explain why some clinically similar lesions follow such divergent biological courses. Taken together, this synthesis provides a meaningful translational insight that may contribute both to the identification of OLs at higher risk of transformation and to the future development of targeted therapeutic strategies and personalized surveillance programs.

## 5. Conclusions

The present scoping review concludes that oral leukoplakia develops early oncogenic mechanisms involved in the malignant transformation of this OPMD, mainly related to sustained proliferation, evasion of growth-suppressor signals, and activation of cell migration and invasion. In addition, proinflammatory processes may facilitate the acquisition of further hallmarks throughout the multistep process of oral carcinogenesis. These findings also expose important evidence gaps regarding relevant hallmarks of cancer, highlighting the need for future research into other distinctive characteristics of cancer cells that may contribute to OL malignant transformation, particularly those related to immune destruction evasion and angiogenesis.

## Figures and Tables

**Table 1:** Summarized study characteristics.

Total Sample	22 Studies
Year of publication	
Range min. (first publication)	2019
Range max.	2025
Study design	
Systematic reviews	8 (36.36%)
Systematic reviews + meta-analysis	14 (63.63%)
Study population	
Oral leukoplakia (OL)	8 (36.36%)
OPMD (including OL)	14 (63.63%)

**Table 2:** Methodological and reporting guidelines, evaluation of risk of bias and quality of evidence.

Study	Year	Population	Study Design	Meta-Analysis	Systematic Reviews Guidelines	Study Protocol (Platform: Registration Code)	Risk of Bias Analysis (Tool)	Quality of Evidence Assessment (System)	Funding/ Conflict of Interest (COI)
Cívico-Ortega et al.	2025	OPMD (including OL)	SR	YES	MOOSE PRISMA Cochrane PRISMA-P	YES (PROSPERO: CRD42024626482)	Quality in Prognosis Studies (QUIPS)	NO	Funding: no COI: none
López-Ansio et al.	2025	OPMD (including OL)	SR	YES	MOOSE PRISMA Cochrane PRISMA-P	YES (CRD42024614644)	Quality in Prognosis Studies (QUIPS)	NO	Funding: no COI: none
Monteiro et al.	2024	OL	SR	YES	PRISMA	YES (PROSPERO: CRD42022329326)	Quality in Prognosis Studies (QUIPS)	NO	Funding: yes COI: none
Huang et al.	2023	OL	SR	YES	PRISMA	YES (INPLASY: INPLASY202250166)	Newcastle–Ottawa Quality Assessment Scale (NOS)	NO	Funding: yes COI: none
Normando et al.	2023	OL	SR	YES	PRISMA PRISMA-P	YES (CRD42020157561)	Joanna Briggs Institute (JBI) tools for Cohort and for Cross-sectional studies	GRADE	Funding: yes COI: none
Benito-Ramal et al.	2023	OPMD (including OL)	SR	YES	PRISMA	NO	Newcastle–Ottawa Quality Assessment Scale (NOS)	NO	Funding: none COI: none
Kumar et al.	2023	OPMD (including OL)	SR	YES	PRISMA	YES (PROSPERO: CRD42020198298)	Newcastle–Ottawa Quality Assessment Scale (NOS)	NO	Funding: none COI: none
Lorenzo-Pouso et al.	2023	OPMD (including OL)	SR	YES	PRISMA-P	YES (PROSPERO: CRD42022355931).	Quality in Prognosis Studies (QUIPS)	NO	Funding: yes COI: none
Ramos-García et al.	2022	OPMD (including OL)	SR	YES	MOOSE PRISMA Cochrane PRISMA-P	YES (CRD42021279108)	Quality in Prognosis Studies (QUIPS)	NO	Funding: none COI: none
Iglesias-Velásquez et al.	2022	OPMD (including OL)	SR	YES	PRISMA	NO	Newcastle–Ottawa Quality Assessment Scale (NOS)	NO	Funding: none COI: none
Monteiro et al.	2021	OL	SR	NO	PRISMA PRISMA-P	YES (CRD42020163464)	Quality in Prognosis Studies (QUIPS)	NO	Funding: yes COI: none
Celentano et al.	2021	OL	SR	NO	PRISMA	NO	Quality in Prognosis Studies (QUIPS)	NO	Funding: yes COI: none
Arroyo et al.	2021	OPDM (including OL)	SR	YES	PRISMA	NO	Quality Assessment of Diagnostic Studies-2 (QUADAS-2)	NO	Funding: none COI: none
Piyarathne et al.	2021	OPDM (including OL)	SR	NO	NO	NO	Newcastle–Ottawa Quality Assessment Scale (NOS)	NO	Funding: none COI: none
Kasradzee et al.	2020	OL	SR	NO	PRISMA	YES (PROSPERO: CRD42015026821)	Cochrane Risk of Bias	NO	Funding: none COI: none
Morais et al.	2020	OPMD (including OL)	SR	NO	PRISMA	NO	REporting recommendations for tumour MARKer prognostic studies (REMARK) statement	NO	Funding: none COI: none
Rivera et al.	2020	OL	SR	NO	NO	YES (PROSPERO: CRD42018086476)	REporting recommendations for tumour MARKer prognostic studies (REMARK) statement	NO	Funding: none COI: none
Ramos García et al.	2019	OPMD (including OL)	SR	YES	MOOSE PRISMA Cochrane PRISMA-P	YES (PROSPERO: CRD42019123753)	Quality in Prognosis Studies (QUIPS)	NO	Funding: none COI: none
Villa et al.	2019	OL	SR	NO	PRISMA	NO	Quality in Prognosis Studies (QUIPS)	NO	Funding: yes COI: none
Saluja et al.	2019	OPMD (including OL)	SR	YES	PRISMA	NO	REporting recommendations for tumour MARKer prognostic studies (REMARK) statement	NO	Funding: none COI: none
Venugopal et al.	2016	OPMD (including OL)	SR	NO	NO	NO	Quality Assessment of Diagnostic Studies	NO	Funding: none COI: none
Smith et al.	2009	OPMD (including OL)	SR	YES	NO	NO	Newcastle–Ottawa Quality Assessment Scale (NOS)	NO	Funding: none COI: none

**Table 3:** Synthesis of evidence from systematic reviews and meta-analyses on cancer hallmarks and associated biomarkers in oral leukoplakia.

**Hallmark of cancer: Sustaining proliferative signaling**	
Cyclin D1	1 study
EGFR	1 study
Ki-67	1 study
**Hallmark of cancer: Evading growth suppressors**	
p53	5 studies
pRb	1 study
p27	1 study
p16	1 study
**Hallmark of cancer: Resisting cell death**	
CYFRA21	1 study
**Hallmark of cancer: Enabling replicative inmortality**	
BMI1	1 study
**Hallmark of cancer: Angiogenesis**	
No evidences	0 studies
**Hallmark of cancer: Activating invasion and metastasis**	
Podoplanin	5 studies
ALDH1	2 studies
CEA	1 study
B-Catenin	1 study
E-Cadherin	1 study
Twist	1 study
MMP9	1 study
**Hallmark of cancer: Deregulating cellular energetics**	
LDH	2 studies
**Hallmark of cancer: Avoiding immune destruction**	
No evidences	0 studies
**Hallmark of cancer: Genome instability and mutation**	
No evidences	0 studies
**Hallmark of cancer: Tumor-promoting inflammation and tumor microenvironment**	
Interleukin 6	3 studies
TNF-α	3 studies
Interleukin 1β	2 studies
CD133	2 studies

**Table 4:** Main results and evidences derived from systematic reviews and meta-analyses.

Biomarker	Study	Year	Population	Design	Key Results
Hallmark: Sustaining proliferative signaling					
EGFR	Cívico-Ortega et al.	2025	OPMD (including OL)	SR + MTA	EGFR upregulation was found to be significantly associated with an elevated malignant transformation risk of OPMD (RR = 2.17, 95% CI = 1.73–2.73, *p* < 0.001). Subgroup analyses demonstrated that OLs also preserved significant results (RR = 1.85, 95% CI = 1.31–2.59; *p* < 0.001).
Ki-67	Normando et al.	2023	OL	SR + MTA	The expression of Ki-67 progressively increased from normal mucosa to OL and oral cancer (*p* < 0.001). Authors suggested that OL patients overexpressing Ki-67 may have a higher risk of developing OSCC. Furthermore, the expression of Ki-67 also increased from hyperplasia to dysplasia in OLs. Ki-67 is the biomarker for which there is the greatest scientific evidence in the malignant transformation of OLs.
Cyclin D1	Ramos-García et al.	2019	OPMD (including OL)	SR + MTA	*CCND1*/cyclin D1 upregulation was significantly associated with higher OPMD malignant transformation risk (RR = 2.31, 95% CI = 1.46 to 3.64, *p* < 0.001). Furthermore, the subgroup mta specifically confirmed significant results for OL ((RR = 1.86, 95% CI = 1.13 to 3.06; *p* = 0.01).
Hallmark: Evading growth suppressors					
pRb	Lopez-Ansio et al.	2025	OPMD (including OL)	SR + MTA	The loss of pRb expression was significantly associated with a higher malignant transformation risk of OPMDs (RR = 1.92, 95% CI = 1.25 to 2.94, *p* = 0.003). The leukoplakia subgroup retained this significant association (*p* = 0.006), being the OPMD where the loss of pRb expression showed the best predictive value for oral cancer development (RR = 2.00, 95% CI = 1.22 to 3.29).
p53	Normando et al.	2023	OL	SR + MTA	The expression of p53 progressively increased from normal mucosa to OL and oral cancer (*p* < 0.005). Authors suggested that OL patients overexpressing p53 may have a higher risk of developing oral cancer. Furthermore, the expression of p53 also increased from hyperplasia to dysplasia in OLs. p53 is the biomarker for which there is the greatest scientific evidence in the malignant transformation of OLs.
	Ramos García et al.	2022	OPMD (including OL)	SR + MTA	p53 overexpression was significantly associated with a higher risk of malignant transformation in patients with OL (RR = 2.22, 95% CI = 1.35–3.64, *p* = 0.002).
	Monteiro et al.	2021	OL	SR	p53 was the most frequently reported protein with significant results in multivariable analyses (*p* < 0.005). However, no stratified statistical data for OL were presented.
	Celentano et al.	2021	OL	SR	The authors identified that the loss of p53 expression is the most promising biomarker for predicting malignant transformation of OLs, acting as an independent predictive factor for progression to oral cancer in the primary-level studies systematically reviewed.
	Smith et al.	2009	OPMD (including OL)	SR + MTA	The risk for cancer progression in p53 positive cases was not significant (RR = 0.96, 95% CI = 0.65 to 1.42; *p* = 0.27). Nevertheless, it should be noted that the results of the present meta-analysis do not derive from a large sample size (n = 6, primary level studies).
p16	Lorenzo-Pouso et al.	2023	OPMD (including OL)	SR + MTA	CDKN2A/p16IN expression was significantly associated with malignant development (RR = 2.01, 95% CI = 1.36 to 2.96; *p* < 0.001), including OL.
p27	Villa et al.	2019	OL	SR	It was identified that the overexpression of p27 is the most promising biomarker for predicting the malignant transformation of OLs. p27 acted as an independent predictive factor for progression to oral cancer in the systematically reviewed primary-level studies.
Hallmark: Resisting cell death					
CYFRA21	Arroyo et al.	2021	OPMD (including OL)	SR + MTA	The salivary expression of CYFRA21 presented significant differences between oral cancer and OPMD, which included OL (MD = 9.31, 95% CI = 9.014 to 9.619; *p* < 0.001).
Hallmark: Enabling replicative immortality					
BMI1	Saluja et al.	2019	OPMD (including OL)	SR + MTA	Bmi1 was considered alongside ALDH1 and CD133. The subgroup meta-analysis for OL showed that this combination of biomarkers was significantly associated with higher risk of malignant transformation (RR = 3.19, 95% CI = 2.55 to 3.98).
Hallmark: Angiogenesis					
No evidences	-	-	-	-	-
Hallmark: Activating invasion and metastasis					
Podoplanin	Monteiro et al.	2024	OPMD (including OL)	SR + MTA	A high expression of podoplanin is significantly associated with an increased risk of oral cancer development in patients with OL (HR = 3.72, 95% CI = 2.40 to 5.76; *p* < 0.001) and could serve as a biomarker for oral malignancy of this OPMD.
	Monteiro et al.	2021	OL	SR	Podoplanin and p53 were the most frequently reported proteins with significant results in multivariable analyses (*p* < 0.005). However, no stratified statistical data for OL were presented.
	Celentano et al.	2021	OL	SR	The authors identified that the overexpression of podoplanin is the most promising biomarker for predicting the malignant transformation of OL. Podoplanin acted as an independent predictive factor for progression to oral cancer in the systematically reviewed primary-level studies.
	Rivera et al.	2020	OL	SR	The overexpression of podoplanin (HR = 8.7, 95% CI = 1.8 to 41.6; *p* = 0.007) in OL was associated with a higher risk of malignant transformation.
	Villa et al.	2019	OL	SR	It was identified that the overexpression of podoplanin is the most promising biomarker for predicting the malignant transformation of OLs. Podoplanin acted as an independent predictive factor for progression to oral cancer in the systematically reviewed primary-level studies.
CEA	Arroyo et al.	2021	OPMD (including OL)	SR + MTA	The salivary expression of CEA presented significant differences between oral cancer and OPMD, which included OL (MD = 25.85, 95% CI = 13.215 to 38.492; *p* < 0.001). Based on these results it was concluded that CEA harbored diagnostic value when differentiating oral cancer from OPMD.
β-catenin	Morais et al.	2020	OPMD (including OL)	SR	The results showed a possible value of β-catenin expression between OL with and without dysplasia (*p* < 0.001).
E-cadherin	Morais et al.	2020	OPMD (including OL)	SR	Significant differences in E-cadherin immuno-expression were observed between normal epithelium and epithelial dysplasia (*p* < 0.001). The results showed a possible value of E-cadherin in the prediction of risk of malignant transformation of oral epithelium.
Twist	Morais et al.	2020	OPMD (including OL)	SR	Significant differences in Twist immuno-expression were observed between normal epithelium and epithelial dysplasia (*p* < 0.001). The results showed a possible value of Twist in the prediction of risk of malignant transformation of oral epithelium.
ALDH1	Rivera et al.	2020	OPMD (including OL)	SR	The overexpression of aldehyde dehydrogenase 1 (ALDH1A1) (HR = 4.2, 95% CI = 2.0 to 8.9; *p* < 0.001) in OL was associated with a higher risk of malignant transformation.
	Saluja et al.	2019	OPMD (including OL)	SR + MTA	ALDH1 was considered alongside Bmi1 and CD133. The subgroup meta-analysis for OL showed that this combination of biomarkers was significantly associated with higher risk of malignant transformation (RR = 3.19, 95% CI = 2.55 to 3.98).
MMP-9	Venugopal et al.	2016	OPMD (including OL)	SR	The biomarker expression in serum was statistically significant between the OL and healthy control group (*p* < 0.001) and showed an increase in the progression from OL to oral cancer (*p* < 0.01).
Hallmark: Deregulating cellular energetics					
LDH	Kumar et al.	2023	OPMD (including OL)	SR + MTA	The salivary lactate dehydrogenase (LDH) levels were higher in patients with OL than in healthy control (*p* < 0.001). This expression biomarker was also higher in patients with head and neck cancer than in patients with OL (*p* < 0.001), thus indicating that LDH could be useful in early detection of oral cancer.
	Iglesias-Velásquez et al.	2022	OPMD (including OL)	SR + MTA	Salivary lactate dehydrogenase (LDH) was significantly higher in OL patients than in healthy control (SMD = 11.67, 95% CI = 1.01 to 22.33; *p* = 0.03), though lower than in oral cancer patients (SMD = 5.62, 95% CI = 2.14 to 9.11; *p* = 0.002).
Hallmark: Avoiding immune destruction					
No evidences	-	-	-	-	-
Hallmark: Genome instability and mutation					
No evidences	-	-	-	-	-
Hallmark: Tumor-promoting inflammation and tumor microenvironment					
IL-6	Huang et al.	2023	OL	SR + MTA	Salivary interleukin 6 (IL-6) levels were higher in OL than in healthy controls (SMD = −1.07, 95% CI = −1.86 to −0.28) and in oral cancer compared to OL (SMD = −1.01, 95% CI = −1.80 to −0.22). These findings suggest a rising trend in IL-6 levels in OL patients.
	Piyarathne et al.	2021	OPMD (including OL)	SR	Salivary interleukin 6 (IL-6) levels were significantly elevated in patients with OL (*p* < 0.05), suggesting an altered immune response. Compared to OL patients and healthy controls, individuals with oral cancer showed markedly higher concentrations of IL-6 (*p* = 0.012). IL-6 expression also increased in parallel with the severity of epithelial dysplasia.
	Kasradzee et al.	2020	OL	SR	Salivary interleukin 6 (IL-6) levels in saliva showed an increase in oral cancer cases in comparison to healthy controls, patients with OL, smokers, and alcohol consumers (*p* ≤ 0.05).
IL-1β	Piyarathne et al.	2021	OPMD (including OL)	SR	Compared to OL patients and healthy controls, individuals with oral cancer showed markedly higher concentrations of IL-1β (*p* < 0.001).
	Kasradzee et al.	2020	OL	SR	Salivary concentrations of salivary Interleukin 1-beta (IL-1β) were significantly elevated in patients with oral cancer compared to those with OL and healthy controls (*p* < 0.005).
TNF-α	Huang et al.	2023	OL	SR + MTA	Tumor necrosis factor alpha (TNF-α) levels had a higher expression in OL than in healthy controls (SMD = −0.83, 95% CI = −1.61 to −0.05) and in oral cancer compared to OL (SMD = −0.86, 95% CI = −1.58 to −0.13). These findings suggest a rising trend in TNF-α levels in OL patients.
	Benito-Ramal et al.	2023	OPMD (including OL)	SR + MTA	The difference in tumor necrosis factor alpha (TNF-α) salivary concentration is statistically significant between the OPMD and healthy control (MD = 21.58, 95% CI = 12.72 to 30.45; *p* < 0.001). However, no stratified results specifically for OL were provided.
	Kasradzee et al.	2020	OL	SR	Salivary tumor necrosis factor alpha (TNF-α) levels were significantly higher in patients with OL compared to healthy control (*p* < 0.005).
CD133	Rivera et al.	2020	OL	SR	The overexpression CD133(HR = 2.9, 95% CI = 1.5 to 5.6; *p* = 0.002) in OL was associated with a higher risk of malignant transformation.
	Saluja et al.	2019	OPMD (including OL)	SR + MTA	CD133 was considered alongside Bmi1 and ALDH1. The subgroup meta-analysis for OL showed that this combination of biomarkers was significantly associated with higher risk of malignant transformation (RR = 3.19, 95% CI = 2.55 to 3.98).

**Table 5:** Main molecular mechanisms involved in OL malignant transformation according to available secondary evidence.

Biomarker	Main Oncogenic Mechanisms Involved in OL Malignant Transformation
EGFR	Activation of important molecular signalling pathways (such as MAPK or PI3K/Akt/mTOR).
Cyclin D1	Regulator of the G1/S transition of the cell cycle and uncontrolled proliferation gain. It can be activated by the above molecular signalling pathways or by *CCND1* gene amplification.
p53	TP53, known as the guardian of the genome, is mutated early, contributing to a decrease in DNA damage repair and resistance to apoptosis promotion.
pRb	pRb is a tumor suppressor protein that inhibits cell proliferation by controlling the cell cycle transition from G1 to S, inducing G1 arrest through the sequestration of E2F transcription factors. This prevents the activation of target genes involved in proliferation.
P16	It is another supressor protein, which exerts a negative regulation of cell proliferation by inhibiting progression through the cell cycle by binding to cyclin-dependent kinases (CDK) 4 or 6 and blocking the action of cyclin D1.
Podoplanin	A transmembrane protein involved in the reorganisation of the actin cytoskeleton and the acquisition of a migratory phenotype through the epithelial–mesenchymal transition phenomenon.
Cytokines	Cytokines and proinflamatory factors (i.e., IL-6, IL-1β, and TNF-α) generate a pro-oncogenic microenvironment that activates oncogenic pathways such as NF-κB, favoring uncontrolled proliferation and the aquisition of other relevants hallmarks of cancer.

## Data Availability

Data sharing not applicable to this article as no datasets were generated or analyzed during the current study.

## Supplementary Materials

The following supporting information can be downloaded at https://www.mdpi.com/article/10.3390/cancers17152427/s1. Table S1. Search strategy for each database, number of results, and execution date; List S1. Detailed search syntax, with thesaurus and free-text terms; List S2. Full-text excluded studies, with reasons.
