# Supplementary material for: Hallmarks of Cancer Expression in Oral Leukoplakia: A Scoping Review of Systematic Reviews and Meta-Analyses
Source: Cancers (Basel). 2025 Jul 22;17(15):2427. doi: 10.3390/cancers17152427 (PMC12346214; doi:10.3390/cancers17152427)
Supplement: Supplementary file 1 [file cancers-17-02427-s001.zip › cancers-3750319-supplementary.pdf]

Supplementary Materials

Hallmarks of cancer expression in oral leukoplakia: a scoping review of systematic reviews and meta-analyses.

Table S1. Search strategy for each database, number of results, and execution date

Database	Query/Search syntax	Results/ Items found	Search time limits
MEDLINE (via PubMed)	Detailed search syntaxis, including thesaurus and free-text terms, is available in supplementary List S1.	18	April 31, 2025
Embase	Detailed search syntaxis, including thesaurus and free-text terms, is available in supplementary List S1.	74	April 31, 2025
Cochrane Database of Systematic Reviews (Cochrane Library)	Detailed search syntaxis, including thesaurus and free-text terms, is available in supplementary List S1.	1	April 31, 2025
Database of Abstracts of Reviews of Effects (DARE)	Detailed search syntaxis, including thesaurus and free-text terms, is available in supplementary List S1.	4	April 31, 2025
Total			97

List S1. Detailed search syntax, with thesaurus and free-text terms

MEDLINE/PubMed

("ErbB Receptors"[Mesh] OR "Genes, erbB-1"[Mesh] OR "epidermal growth factor receptor"[all fields] OR "egfr"[all fields] OR erbb*[all fields] OR "EGF Family of Proteins"[Mesh] OR "Epidermal Growth Factor"[Mesh] OR "epidermal growth factor"[all fields] OR "egf"[all fields] OR "Genes, erbB-2"[Mesh] OR "Receptor, ErbB-2"[Mesh] OR "Receptor, ErbB-3"[Mesh] OR "neu"[all fields] OR "erbb2"[all fields] OR "erbb3"[all fields] OR "erbb4"[all fields] OR "cerbb2"[all fields] OR "cerbb3"[all fields] OR "cerbb4"[all fields] OR "her2"[all fields] OR "her3"[all fields] OR "her4"[all fields] OR "cyclin d1"[MeSH] OR ("cyclin"[All Fields] AND "d1"[All Fields]) OR "cyclin d1"[All Fields] OR "cyclind1"[All Fields] OR "ccnd1"[All Fields] OR "ccnd 1"[All Fields] OR "Genes, ras"[Mesh] OR "ras Proteins"[Mesh] OR "ras"[All Fields] OR "hras"[All Fields] OR "kras"[All Fields] OR "nras"[All Fields] OR "Phosphatidylinositol 3-Kinases"[Mesh] OR "pi3k"[All Fields] OR "akt"[All Fields] OR "mtor"[All Fields] OR "pten"[All Fields] OR "NF-kappa B"[Mesh] OR "I-kappa B Kinase"[Mesh] OR "nuclear factor kappa b"[All Fields] OR "nf kappa b"[All Fields] OR "nfkb"[All Fields] OR "i kappa b kinase"[All Fields] OR "ikk"[All Fields] OR "STAT Transcription Factors"[Mesh] OR "Janus Kinases"[Mesh] OR "Signal transducers and activators of transcription"[All Fields] OR "stat"[All Fields] OR "stat3"[All Fields] OR "stat5"[All Fields] OR "janus"[All Fields] OR "jak"[All Fields] OR "jak1"[All Fields] OR "jak2"[All Fields] OR "Mitogen-Activated Protein Kinase Kinases"[Mesh] OR "MAP Kinase Signaling System"[Mesh] OR "Proto-Oncogene Proteins B-raf"[Mesh] OR "mitogen activated protein kinase"[All Fields] OR "mapk"[All Fields] OR "mapkk"[All Fields] OR "mapkkk"[All Fields] OR "braf"[All Fields] OR "mek"[All Fields] OR "erk"[All Fields] OR "Retinoblastoma[Mesh]" OR "retinoblastoma"[All Fields] OR "rb"[All Fields] OR "prb"[All Fields] OR "osrc"[All Fields] OR "pp110"[All Fields] OR "p105-Rb"[All Fields] OR "ppp1r130"[All Fields] OR "p110-rb1"[All Fields] OR "Cyclin-Dependent Kinase Inhibitor p16"[Mesh] OR "p16"[All Fields] OR "cdkn2a"[All Fields] OR "ink4a"[All Fields] OR "Cyclin-Dependent Kinase Inhibitor p15"[Mesh] OR "p15"[All Fields] OR "cdkn2b"[All Fields] OR "ink4b"[All Fields] OR "Cyclin-Dependent Kinase Inhibitor p18"[Mesh] OR "p18"[All Fields] OR "cdkn2c"[All Fields] OR "ink4c"[All Fields] OR "p19"[All Fields] OR "cdkn2d"[All Fields] OR "ink4d"[All Fields] OR "Cyclin-Dependent Kinase Inhibitor p21"[Mesh] OR "p21"[All Fields] OR "cdkn1a"[All Fields] OR "Cyclin-Dependent Kinase Inhibitor p27"[Mesh] OR "p27"[All Fields] OR "cdkn1b"[All Fields] OR "Cyclin-Dependent Kinase Inhibitor p57"[Mesh] OR "p57"[All Fields] OR "cdkn1c"[All Fields] OR "Tumor Suppressor Protein p53"[MeSH] OR "Genes, p53"[MeSH] OR "p53"[All Fields] OR "tp53"[All Fields] OR "Neurofibromatosis 2"[MeSH] OR "Neurofibromin 2"[MeSH] OR "neurofibromatosis 2"[All Fields] OR "nf2"[All Fields] OR "Neurofibromin 2"[All Fields] OR "merlin"[All Fields] OR "lkb1"[All Fields] OR "Transforming Growth Factor beta"[Mesh] OR "transforming growth factor beta 1"[All Fields] OR "transforming growth factor beta 2"[All Fields] OR "transforming growth factor beta 3"[All Fields] OR "transforming growth factor beta"[All Fields] OR "tgfb" OR "tgf beta"[All Fields] OR "Caspases"[Mesh] OR "Caspase 1"[Mesh] OR "Caspase 2"[Mesh] OR "Caspase 3"[Mesh] OR "Caspase 6"[Mesh] OR "Caspase 7"[Mesh] OR "Caspase 8"[Mesh] OR "Caspase 9"[Mesh] OR "Caspase 10"[Mesh] OR "Caspase 12"[Mesh] OR "Caspase 14"[Mesh] OR caspase*[All Fields] OR "Genes, bcl-2"[Mesh] OR "Proto-Oncogene Proteins c-bcl-2"[Mesh] OR "bcl-X Protein"[Mesh] OR "bcl-2 Homologous Antagonist-Killer Protein"[Mesh] OR "bcl2"[All Fields] OR "bax"[All Fields] OR "bclx"[All Fields] OR "bak"[All Fields] OR "bclw"[All Fields] OR "mcl1"[All Fields] OR "Cytochrome c Group"[Mesh] OR "cytochrome c"[All Fields] OR "cyt c"[All Fields] OR "cyc"[All Fields] OR "NOXA1"[Mesh] OR "NADPH oxidase activator 1"[Mesh] OR "noxa"[All Fields] OR "nadph oxidase activator 1"[All Fields] OR "puma"[All Fields] OR "bbc3"[All Fields] OR "jfy1"[All Fields] OR "bcl2 homology region 3 bh3 only"[All Fields] OR "bh3 only"[All Fields] OR "bim"[All Fields] OR "Autophagy"[Mesh] OR "autophagy"[All Fields] OR "Beclin-1"[Mesh] OR "beclin-1"[All Fields] OR "becn1"[All Fields] OR "atg6"[All Fields] OR "vps30"[All Fields] OR "Necrosis"[Mesh] OR "necrosis"[All Fields] OR "Telomerase"[Mesh] OR "telomerase"[All Fields] OR "tert"[All Fields] OR "Vascular Endothelial Growth Factors"[Mesh] OR ("vascular"[All Fields] AND "endothelial"[All Fields] AND "growth"[All Fields] AND factor*[All Fields]) OR "vegf" [All Fields] OR "Receptors, Vascular Endothelial Growth Factor"[Mesh] OR ("vascular"[All Fields] AND "endothelial"[All Fields] AND "growth"[All Fields] AND factor*[All Fields] AND receptor*[All Fields]) OR "VEGFR"[All Fields] OR "tsp1"[All Fields] OR "tsp"[All Fields] OR "thrombospondin 1"[All Fields] OR "thbs1"[All Fields] OR "thbs"[All Fields] OR "Fibroblast Growth Factors"[Mesh] OR ("fibroblast"[All Fields] AND "growth"[All Fields] AND factor*[All Fields]) OR "fgf" [All Fields] OR "plasmin"[All Fields] OR "angiostatin" [All Fields] OR "Endostatins"[Mesh] OR endostatin*[All Fields] OR "collagen type 18"[All Fields] OR "Pericytes"[Mesh] OR pericyt*[All Fields] OR "rouget cells"[All Fields] OR "Cadherins"[Mesh] OR "e-cadherin"[All Fields] OR cadherin*[All Fields] OR "cd324"[All Fields] OR "cdh1"[All Fields] OR "n-cadherin"[All Fields] OR "CD325"[All Fields] OR "CDH2"[All Fields] OR "Epithelial-Mesenchymal Transition"[Mesh] OR ("epithelial"[All Fields] AND "mesenchymal"[All Fields] AND "transition"[All Fields]) OR "emt"[All Fields] OR snail*[All Fields] OR "slugh2"[All Fields] OR "sna"[All Fields] OR "snah"[All Fields] OR "slug"[All Fields] OR "slugh1"[All Fields] OR "Twist-Related Protein 1"[Mesh] OR "Twist Transcription Factors"[Mesh] OR twist*[all fields] or "bhlh"[all fields] or "scs"[all fields] or "h-twist"[all fields] or "bpes2"[all fields] or "bhlha38"[all fields] or "crs1"[all fields] OR "zeb1"[all fields] OR "zeb2"[all fields] OR "zinc finger E-box binding homeobox 1"[all fields] OR "zinc finger E-box binding homeobox 2"[all fields] OR "tcf8"[all fields] OR "ppcd3"[all fields] OR "bzp"[all fields] OR "zeb"[all fields] OR "areb6"[all fields] OR "nil-2-a"[all fields] OR "zfhep"[all fields] OR "zfhx1a"[all fields] OR "fecd6"[all fields] OR "zfhx1b"[all fields] OR "kiaa0569"[all fields] OR "sip1"[all fields] OR "caretaker"[all fields] OR brca*[all fields] OR "rad51"[all fields] OR "rnf53"[all fields] OR

"brcc1"[all fields] OR "ppp1r53"[all fields] OR "fancs"[all fields] OR "fancd1"[all fields] OR "facd"[all fields] OR "fancd"[all fields] OR "rad51a"[all fields] OR "reca"[all fields] OR "hsrad51"[all fields] OR "hst16930"[all fields] OR "brcc5"[all fields] OR "fancr"[all fields] OR "atm"[all fields] OR "ata"[all fields] OR "atdc"[all fields] OR "atc"[all fields] OR "atd"[all fields] OR "tel1"[all fields] OR "telo1"[all fields] OR "DNA Copy Number Variations"[Mesh] OR "Gene Amplification"[Mesh] OR "Sequence Deletion"[Mesh] OR "Glucose Transporter Type 1"[Mesh] OR "glucose transporter type"[All Fields] OR "glut1"[All Fields] OR "hypoxia inducible factor"[All Fields] OR "hif1a"[All Fields] OR "hif-1alpha"[All Fields] OR "pasd8"[All Fields] OR "bHLHe78"[All Fields] OR "HIF2A"[All Fields] OR "HIF-1 alpha-like factor"[All Fields] OR "mop2"[All Fields] OR "pasd2"[All Fields] OR "hlf"[All Fields] OR "bhLhe73"[All Fields] OR "Warburg Effect, Oncologic"[Mesh] OR "warburg"[All Fields] OR "aerobic glycolysis"[All Fields] OR "Isocitrate Dehydrogenase"[Mesh] OR "isocitrate dehydrogenase1"[All Fields] OR "isocitrate dehydrogenase2"[All Fields] OR "idh"[All Fields] OR "idh1"[All Fields] OR "idh2"[All Fields] OR "Tumor Escape"[Mesh] OR (("evading"[All Fields] OR "evasion"[All Fields] OR "escape"[All Fields]) AND "immune"[All Fields]) OR "Neoplastic Stem Cells"[Mesh] OR "cancer stem cells"[All Fields] OR "csc"[All Fields] OR "Endothelial Cells"[Mesh] OR endothel*[All Fields] OR "Angiopoietin-1"[Mesh] OR "ang-1"[All Fields] OR "angiopoietin 1"[All Fields] OR "kiaa0003"[All Fields] OR "angpt1"[All Fields] OR "Receptor, TIE-2"[Mesh] OR "tie2"[All Fields] OR "vmcm"[All Fields] OR "vmcm1"[All Fields] OR "cd202b"[All Fields] OR "tek"[All Fields] OR "angiopoietin-1 receptor"[All Fields] OR "Receptors, Platelet-Derived Growth Factor"[Mesh] OR "pdgf"[All Fields] OR "platelet derived growth factor receptor"[All Fields] OR "Cathepsins"[Mesh] OR cathepsin*[All Fields] OR "heparanase"[All Fields] OR hpse*[All Fields] OR "Myeloid-Derived Suppressor Cells"[Mesh] OR mdsc*[All Fields] OR "Matrix Metalloproteinases"[Mesh] OR "matrix metallopeptidase"[All Fields] OR "mmp"[All Fields] OR "mmt"[All Fields] OR "mmp1"[All Fields] OR "mmp2"[All Fields] OR "mmp3"[All Fields] OR "mmp7"[All Fields] OR "mmp8"[All Fields] OR "mmp9"[All Fields] OR "mmp10"[All Fields] OR "mmp11"[All Fields] OR "mmp12"[All Fields] OR "mmp13"[All Fields] OR "mmp14"[All Fields] OR "mmp15"[All Fields] OR "mmp16"[All Fields] OR "mmp17"[All Fields] OR "mmp18"[All Fields] OR "mmp19"[All Fields] OR "mmp20"[All Fields] OR "mmp21"[All Fields] OR "mmp24"[All Fields] OR "mmp25"[All Fields] OR "mmp26"[All Fields] OR "mmp27"[All Fields] OR "mmp28"[All Fields] OR "mmp23B"[All Fields] OR "Chemokines"[Mesh] OR "ccl"[All Fields] OR "cxcl"[All Fields] OR "Cytokines"[Mesh] OR interferon*[All Fields] OR interleukin*[All Fields] OR lymphokines*[All Fields] OR monokine*[All Fields] OR oncostatin*[All Fields] OR osteopontin*[All Fields] OR "tumor necrosis factor"[All Fields] OR "tnf-α"[All Fields] OR "macrophages"[Mesh] OR macrophage*[All Fields] OR monocyte*[All Fields] OR histiocyte*[All Fields] OR "Neutrophils"[Mesh] OR neutrophil*[All Fields] OR "polymorphonuclear"[All Fields] OR histiocyte*[All Fields] OR "Cancer-Associated Fibroblasts"[Mesh] OR ("cancer"[All Fields] AND "associated"[All Fields] AND "fibroblasts"[All Fields]) OR "a-sma"[All Fields] OR ("alpha"[All Fields] AND "smooth"[All Fields] AND "muscle"[All Fields] AND "actin"[All Fields]) OR ("stem cells"[All Fields] NOT "cancer stem cells"[All Fields]) OR "hoxa5"[All Fields] OR "homeobox a5"[All Fields] OR "hox1c"[All Fields] OR "hox1"[All Fields] OR "Smad4 Protein"[Mesh] OR "smad4"[All Fields] OR "smad"[All Fields] OR "madh4"[All Fields] OR "mitf"[All Fields] OR "melanocyte inducing transcription factor"[All Fields] OR "ws2a"[All Fields] OR "ws2"[All Fields] OR "bhlhe32"[All Fields] OR "Activating Transcription Factor 2"[Mesh] OR "atf2"[All Fields] OR "activating transcription factor 2"[All Fields] OR "cre-binding protein 1"[All Fields] OR "Retinoic Acid Receptor alpha"[Mesh] OR "rar alpha"[All Fields] OR "rar-a"[All Fields] OR "RUNX1 Translocation Partner 1 Protein"[Mesh] OR runx*[All Fields] OR "aml1-eto"[All Fields] OR "aml1"[All Fields] OR "cbfa2"[All Fields] OR "SOX Transcription Factors"[Mesh] OR "SOXE Transcription Factors"[Mesh] OR "sox10"[All Fields] OR "sry-box transcription factor 10"[All Fields] OR "dom"[All Fields] OR "ws4"[All Fields] OR "ws2e"[All Fields] OR "alpha-ketoglutarate"[All Fields] OR "αkg"[All Fields] OR "a-KG"[All Fields] OR "d-2-hydroxygluterate"[All Fields] OR "d2hg"[All Fields] OR "pancreas associated transcription factor 1a"[All Fields] OR "ptf1a"[All Fields]) AND ("Leukoplakia, Oral"[MeSH] OR "oral leukoplakia" [All Fields]) AND ("Meta-Analysis"[pt] OR "meta-analysis"[tiab] OR "Systematic Review"[pt] OR "systematic review"[tiab])

Embase

('epidermal growth factor receptor'/exp OR 'erbB-1' OR 'epidermal growth factor receptor' OR 'EGFR' OR 'erbb*' OR 'epidermal growth factor derivative'/exp OR 'epidermal growth factor' OR 'egf' OR 'epidermal growth factor receptor 2'/exp OR 'erbb2' OR 'neu' OR 'epidermal growth factor receptor 3'/exp OR 'erbb3' OR 'erbb4' OR 'cerbb2' OR 'cerbb3' OR 'cerbb4' OR 'her2' OR 'her3' OR 'her4' OR 'cyclin d1'/exp OR 'cyclin d1' OR 'cyclind1' OR 'ccnd1' OR 'ccnd 1' OR 'ras' OR 'hras' OR 'kras' OR 'nras' OR 'Akt signaling'/exp OR 'pi3k' OR 'akt' OR 'mtor' OR 'pten' OR 'NF kB signaling'/exp OR 'nuclear factor kappa b' OR 'nuclear factor kappa b' OR 'nf kappa b' OR 'nfkb' OR 'i kappa b kinase' OR 'ikk' OR 'JAK-STAT signaling'/exp OR 'stat' OR 'Signal transducers and activators of transcription' OR 'stat3' OR 'stat5' OR 'janus' OR 'jak' OR 'jak1' OR 'jak2' OR 'MAPK signaling'/exp OR 'mitogen activated protein kinase' OR 'mapk' OR 'mapkk' OR 'mapkkk' OR 'braf' OR 'mek' OR 'erk' OR 'retinoblastoma'/exp OR 'rb' OR 'prb' OR 'osrc' OR 'pp110' OR 'p105-Rb' OR 'PPP1R130' OR 'p110-RB1' OR 'cyclin dependent kinase inhibitor 2A'/exp OR 'p16' OR 'cdkn2a' OR 'ink4a' OR 'cyclin dependent kinase inhibitor 2B'/exp OR 'p16' OR 'cdkn2b' OR 'ink4b' OR 'cyclin dependent kinase inhibitor 2C'/exp OR 'p18' OR 'cdkn2c' OR 'ink4c' OR 'cyclin dependent kinase inhibitor 2D'/exp OR 'p19' OR 'cdkn2d' OR 'ink4d' OR 'p21' OR 'cdkn1a' OR 'p27' OR 'cdkn1b' OR 'p57' OR 'cdkn1c' OR 'protein p53'/exp OR 'p53 signaling'/exp OR 'p53' OR 'TP53' OR 'neurofibromatosis type 2'/exp OR 'merlin'/exp OR 'neurofibromaatosis 2' OR 'nf2' OR 'neurofibrin 2' OR 'lkb1' OR 'transforming growth factor beta'/exp OR 'transforming growth

factor beta 1' OR 'transforming growth factor beta 2' OR 'transforming growth factor beta 3' OR 'transforming growth factor beta 4' OR ' tgfb' OR ' tgf beta' OR 'caspase'/exp OR 'Caspase 1' OR 'Caspase 2' OR 'Caspase 3' OR 'Caspase 6' OR 'Caspase 7' OR 'Caspase 8' OR 'Caspase 9' OR 'Caspase 10' OR 'Caspase 12' OR 'Caspase 14' OR 'caspase*' OR 'bcl-2' OR 'bax' OR 'bak' OR 'bclw' OR 'mcl1' OR 'cytochrome c'/exp OR 'cyt c' OR 'cyc' OR 'noxa1' OR 'NADPH oxidase activator 1' OR 'puma' OR 'jfy1' OR 'bh3' OR 'bim' OR 'autophagy' OR 'beclin-1' OR 'becn1' OR 'atg6' OR 'vps30' OR 'necrosis'/exp OR 'telomerase'/exp OR 'tert' OR 'vasculotropin'/exp OR 'vegf' OR 'vasculotropin receptor'/exp OR 'vegfr' OR 'tsp1' OR 'tsp' OR 'thrombospondin 1' OR 'thbs1' OR 'thbs' OR 'fibroblast growth factor'/exp OR 'fgf' OR 'plasmin' OR 'angiostatin' OR 'endostatin'/exp OR 'collagen type 18' OR 'pericyte'/exp OR 'pericyt*' OR 'rouget cells' OR 'cadherin'/exp OR 'e-cadherin' OR 'cd324' OR 'cdh1' OR 'n-cadherin' OR 'cd325' OR 'cdh2' OR 'epithelial mesenchymal transition'/exp OR 'emt' OR 'snail*' OR 'slug' OR 'slugh1' OR 'slugh2' OR 'sna' OR 'snah' OR 'twist' OR 'twist*' OR 'bhlh' OR 'scs' OR 'h-twist' OR 'bpes2' OR 'bhlha38' OR 'crs1' OR 'zeb1' OR 'zeb2' OR ' zinc finger E-box binding homeobox 1' OR 'zinc finger E-box binding homeobox 2' OR 'tcf8' OR 'ppcd3' OR 'bzp' OR 'zeb' OR 'areb6' OR 'nil-2-a' OR 'zfhep' OR ' zfhx1a' OR 'fecd6' OR 'zfh1b' OR 'kiaa0569' OR 'sip1' OR 'caretaker' OR 'brca*' OR 'rad51' OR 'rnf53' OR 'brcc1' OR 'ppp1r53' OR 'fancs' OR 'fancd1' OR 'facd' OR 'fancd' OR 'rad51a' OR 'reca' OR 'hsrad51' OR 'hst16930' OR 'brcc5' OR 'fancr' OR 'atm' OR 'ata' OR 'atdc' OR 'atc' OR 'atd' OR 'tel1' OR 'telo1' OR 'DNA Copy Number Variations' OR 'gene amplification' OR 'sequence deletion' OR 'glucose transporter type 1' OR 'glut1' OR 'hypoxia inducible factor' OR 'hif1a' OR 'hif-1alpha' OR 'pasd8' OR 'bhlhe78' OR 'hif2a' OR 'hif-1 alpha-like factor' OR 'mop2' OR 'pasd2' OR 'hlf' OR 'bhlhe73' OR 'warburg' OR 'aerobic glycolysis' OR 'isocitrate deshydrogenase*' OR 'idh' OR 'idh1' OR 'idh2' OR 'tumor escape' OR 'immune' OR 'stem cell*' OR 'csc' OR 'endothel*' OR 'angiopoietin*' OR 'ang-1' OR 'kiaa0003' OR 'angpt1' OR 'tie2' OR 'vmcm*' OR 'cd202b' OR 'tek' OR ' Platelet-Derived Growth Factor' OR 'PDGF*' OR 'cathepsin*' OR 'heparanase' OR 'hpse*' OR 'Myeloid-Derived Suppressor Cells' OR 'mdsc*' OR 'matrix metalloproteinase'/exp OR 'mmp' OR 'matrix metallopeptidase' OR 'mmt' OR 'mmp1' OR 'mmp2' OR 'mmp3' OR 'mmp7' OR 'mmp8' OR 'mmp9' OR 'mmp10' OR 'mmp11' OR 'mmp12' OR 'mmp13' OR 'mmp14' OR 'mmp15' OR 'mmp16' OR 'mmp17' OR 'mmp18' OR 'mmp19' OR 'mmp20' OR 'mmp21' OR 'mmp24' OR 'mmp25' OR 'mmp26' OR 'mmp27' OR 'mmp28' OR 'mmp23b' OR 'chemokine'/exp OR 'ccl' OR 'cxcl' OR 'cytokine'/exp OR 'interferon*' OR 'interleukin*' OR 'lymphokine*' OR 'monokine*' OR 'oncostatin*' OR 'osteopontin*' OR 'tumor necrosis factor' OR 'macrophage*' OR 'monocyte*' OR 'histiocyte' OR 'neutrophil*' OR 'polymorphonuclear' OR 'histiocyte*' OR 'cancer associated fibroblast'/exp OR 'a-sma' OR 'alpha smooth muscle actin' OR 'stem cell*' OR 'hoxa5' OR 'homeobox a5' OR 'hox1c' OR 'hox1' OR 'smad*' OR 'madh4' OR 'mitf' OR 'melanocyte inducing transcription factor' OR 'ws2a' OR 'ws2' OR 'bHLHe32' OR 'Activating Transcription Factor 2' OR 'atf2' OR 'CRE-Binding Protein 1' OR 'Retinoic Acid Receptor alpha' OR 'Rar alpha' OR 'rar-a' OR 'runx*' OR 'AML1-ETO' OR 'AML1' OR 'CBFA2' OR 'sox' OR 'soxe' OR 'sox10' OR 'SRY-box transcription factor 10' OR 'dom' OR 'ws4' OR 'ws2e' OR 'alpha-ketoglutarate' OR 'αKG' OR 'a-KG' OR 'D-2-hydroxygluterate' OR 'd2hg' OR 'pancreas associated transcription factor 1a' OR 'PTF1a') AND (('leukoplakia'/exp OR 'leukoplakia') AND ('mouth'/exp OR 'oral')) AND ('systematic review':ti,ab OR [systematic review]/lim OR 'meta-analysis':ti,ab OR [meta analysis]/lim)

Cochrane Library

MeSH descriptor: [leukoplakia, oral] explode all trees

DARE

MeSH DESCRIPTOR Leukoplakia, Oral EXPLODE ALL TREES

List S2. Full-text excluded studies, with reasons

Off topic (*n* = 16)

Abdul, N. S., Rashdan, Y., Alenezi, N., Alenezi, M., Mohsin, L., & Hassan, A. (2024). Association Between Oral Microbiota and Oral Leukoplakia: A Systematic Review. Cureus, 16(1), e52095.

Aguirre-Urizar, J. M., Lafuente-Ibáñez de Mendoza, I., & Warnakulasuriya, S. (2021). Malignant transformation of oral leukoplakia: Systematic review and meta-analysis of the last 5 years. *Oral diseases*, *27*(8), 1881–1895.

Bhattarai, B. P., Singh, A. K., Singh, R. P., Chaulagain, R., Søland, T. M., Hasséus, B., & Sapkota, D. (2024). Recurrence in Oral Leukoplakia: A Systematic Review and Meta-analysis. *Journal of dental research*, *103*(11), 1066–1075.

De Freitas Silva, B. S., Batista, D. C. R., de Souza Roriz, C. F., Silva, L. R., Normando, A. G. C., Dos Santos Silva, A. R., Silva, M. A. G., & Yamamoto-Silva, F. P. (2021). Binary and WHO dysplasia grading systems for the prediction of malignant transformation of oral leukoplakia and erythroplakia: a systematic review and meta-analysis. *Clinical oral investigations*, *25*(7), 4329–4340.

De Pauli Paglioni, M., Migliorati, C. A., Schausltz Pereira Faustino, I., Linhares Almeida Mariz, B. A., Oliveira Corrêa Roza, A. L., Agustin Vargas, P., Franco Paes Leme, A., Bianca Brandão, T., Prado Ribeiro, A. C., Ajudarte Lopes, M., & Santos-Silva, A. R. (2020). Laser excision of oral leukoplakia: Does it affect recurrence and malignant transformation? A systematic review and meta-analysis. *Oral oncology*, *109*, 104850.

Grajewski, S., Quarcoo, D., Uibel, S., Scutaru, C., Groneberg, D., & Spallek, M. (2010). Eine szientometrische Analyse der Leukoplakie und Erythroplakie [A scientometric analysis of leukoplakia and erythroplakia]. *Laryngo- rhino- otologie*, *89*(4), 210–215.

Guan, J. Y., Luo, Y. H., Lin, Y. Y., Wu, Z. Y., Ye, J. Y., Xie, S. M., & Li, J. (2023). Malignant transformation rate of oral leukoplakia in the past 20 years: A systematic review and meta-analysis. *Journal of oral pathology & medicine : official publication of the International Association of Oral Pathologists and the American Academy of Oral Pathology*, *52*(8), 691–700.

Iocca, O., Sollecito, T. P., Alawi, F., Weinstein, G. S., Newman, J. G., De Virgilio, A., Di Maio, P., Spriano, G., Pardiñas López, S., & Shanti, R. M. (2020). Potentially malignant disorders of the oral cavity and oral dysplasia: A systematic review and meta-analysis of malignant transformation rate by subtype. *Head & neck*, *42*(3), 539–555.

Paglioni, M. P., Khurram, S. A., Ruiz, B. I. I., Lauby-Secretan, B., Normando, A. G., Ribeiro, A. C. P., Brandão, T. B., Palmier, N. R., Lopes, M. A., da Silva Guerra, E. N., Meleti, M., Migliorati, C. A., Carvalho, A. L., de Matos, L. L., Kowalski, L. P., & Santos-Silva, A. R. (2022). Clinical predictors of malignant transformation and recurrence in oral potentially malignant disorders: A systematic review and meta-analysis. *Oral surgery, oral medicine, oral pathology and oral radiology*, *134*(5), 573–587.

Patil, S., Arakeri, G., Alamir, A. W. H., Patil, S., Awan, K. H., Baeshen, H., Raj, T., Fonseca, F. P., & Brennan, P. A. (2020). Is toombak a risk factor for oral leukoplakia and oral squamous cell carcinoma ? A systematic review. *Journal of oral pathology & medicine : official publication of the International Association of Oral Pathologists and the American Academy of Oral Pathology*, *49*(2), 103–109

Pimenta-Barros, L. A., Ramos-García, P., González-Moles, M. Á., Aguirre-Urizar, J. M., & Warnakulasuriya, S. (2024). Malignant transformation of oral leukoplakia: Systematic review and comprehensive meta-analysis. *Oral diseases*, 10.1111/odi.15140. Advance online publication.

Pinto, A. C., Caramês, J., Francisco, H., Chen, A., Azul, A. M., & Marques, D. (2020). Malignant transformation rate of oral leukoplakia-systematic review. *Oral surgery, oral medicine, oral pathology and oral radiology*, *129*(6), 600–611.e2.

Roza, A. L. O. C., Kowalski, L. P., William, W. N., Jr, de Castro, G., Jr, Chaves, A. L. F., Araújo, A. L. D., Ribeiro, A. C. P., Brandão, T. B., Lopes, M. A., Vargas, P. A., Santos-Silva, A. R., & Latin American Cooperative Oncology Group–Brazilian Group of Head and Neck Cancer (2021). Oral leukoplakia and erythroplakia in young patients: a systematic review. *Oral surgery, oral medicine, oral pathology and oral radiology*, *131*(1), 73–84.

Shiu, M. N., & Chen, T. H. (2003). Intervention efficacy and malignant transformation to oral cancer among patients with leukoplakia (Review). *Oncology reports*, *10*(6), 1683–1692.

Warnakulasuriya, S., & Ariyawardana, A. (2016). Malignant transformation of oral leukoplakia: a systematic review of observational studies. *Journal of oral pathology & medicine : official publication of the International Association of Oral Pathologists and the American Academy of Oral Pathology*, *45*(3), 155–166.

Zhang, C., Li, B., Zeng, X., Hu, X., & Hua, H. (2023). The global prevalence of oral leukoplakia: a systematic review and meta-analysis from 1996 to 2022. *BMC oral health*, *23*(1), 645.

Non-systematic review (*n* = 3)

Abatti-Chiamulera, M. M., Zancan, C. B., Remor, A. P., Cordeiro, M. F., Gleber-Netto, F. O., & Baptistella, A. R. (2021). Salivary cytokines as biomarkers of oral cancer: a systematic review and meta-analysis. BMC cancer, 21(1), 205.

Yap, T., Pruthi, N., Seers, C., Belobrov, S., McCullough, M., & Celentano, A. (2020). Extracellular Vesicles in Oral Squamous Cell Carcinoma and Oral Potentially Malignant Disorders: A Systematic Review. International journal of molecular sciences, 21(4), 1197.

Zhang, E., Cui, Z., Xu, Z., Duan, W., Huang, S., Tan, X., Yin, Z., Sun, C., & Lu, L. (2013). Association between polymorphisms in ERCC2 gene and oral cancer risk: evidence from a meta-analysis. BMC cancer, 13, 594

No oral cavity (*n* = 1)

Wan, P., Ongkasuwan, J., Martinez, J., Sandulache, V., Deng, D., Jiang, J., Sikora, A., & Altman, K. W. (2021). Biomarkers for Malignant Potential in Vocal Fold Leukoplakia: A State of the Art Review. Otolaryngology--head and neck surgery : official journal of American Academy of Otolaryngology-Head and Neck Surgery, 164(4), 751–758.

Commentary (n = 1)

Chasma, F., Pedr King, R., & Ker, S. Y. (2022). Are there diagnostic alternatives to histopathology in detecting oral cancer?. Evidence-based dentistry, 23(1), 24–25. 19.
